# Hypofibrinogenemia is associated with a high degree of risk in infectious diseases: a post-hoc analysis of post-marketing surveillance of patients with disseminated intravascular coagulation treated with thrombomodulin alfa

**DOI:** 10.1186/s12959-021-00264-z

**Published:** 2021-02-25

**Authors:** Kazuo Kawasugi, Hideo Wada, Goichi Honda, Noriaki Kawano, Toshimasa Uchiyama, Seiji Madoiwa, Naoki Takezako, Kei Suzuki, Yoshinobu Seki, Takayuki Ikezoe, Toshiaki Iba, Kohji Okamoto

**Affiliations:** 1 Faculty of Medical Technology, Teikyo University, Tokyo, Japan; 2 Department of General Medicine, Mie Prefectural General Medical Center, Yokkaichi, Japan; 3 Department of Medical Affairs, Asahi Kasei Pharma Corporation, Tokyo, Japan; 4 Department of Internal Medicine, Miyazaki Prefectural Miyazaki Hospital, Miyazaki, Japan; 5 Department of Laboratory Medicine, National Hospital Organization Takasaki General Medical Center, Takasaki, Japan; 6 Department of Clinical Laboratory Medicine, Tokyo Saiseikai Central Hospital, Tokyo, Japan; 7 Department of Hematology, National Hospital Organization Disaster Medical Center, Tokyo, Japan; 8 Emergency and Critical Care Center, Mie University Hospital, Tsu, Japan; 9 Department of Hematology, Uonuma Institute of Community Medicine, Niigata University Medical and Dental Hospital, Niigata, Japan; 10 Department of Hematology, Fukushima Medical University, Fukushima, Japan; 11 Department of Emergency and Disaster Medicine, Juntendo University Graduate School of Medicine, Tokyo, Japan; 12 Department of Surgery, Center for Gastroenterology and Liver Disease, Kitakyushu City Yahata Hospital, Fukuoka, Japan

**Keywords:** Disseminated intravascular coagulation, Hypofibrinogenemia, Thrombomodulin, Post-marketing surveillance, Infectious disease, Mortality

## Abstract

**Background:**

In patients with infectious diseases, disseminated intravascular coagulation (DIC) is often diagnosed without the fibrinogen value. The relationship between hypofibrinogenemia and outcomes of DIC in infectious diseases has thus remained unclear.

**Methods:**

We analyzed 3204 patients who received with thrombomodulin alfa (TM-α) for DIC and suspected DIC. Hypofibrinogenemia was defined by a fibrinogen level < 1.5 g/L.

**Results:**

Hypofibrinogenemia was observed in 10.3% of patients with infectious diseases. The frequencies of both bleeding and organ failure symptoms, and the scores for organ failure or the DIC diagnostic criteria were significantly higher in infectious disease patients with hypofibrinogenemia, suggesting that in patients with infectious diseases, hypofibrinogenemia is associated with more progressive and severe DIC. Although the 28-day survival rate and the DIC resolution rate were both significantly lower for infectious disease patients with DIC with hypofibrinogenemia than for those without hypofibrinogenemia, this difference was not observed in DIC patients with hematological diseases.

**Conclusions:**

Hypofibrinogenemia among infectious disease patients with DIC may reflect increased consumption of fibrinogen due to accelerated coagulation reactions, while hypofibrinogenemia among hematological disease patients with DIC may be caused by fibrinogenolysis due to hyperfibrinolysis, and frequently results in bleeding and multiple-organ failure.

**Supplementary Information:**

The online version contains supplementary material available at 10.1186/s12959-021-00264-z.

## Introduction

Disseminated intravascular coagulation (DIC), which occurs in association with various underlying diseases, such as sepsis, hematological malignancy, solid tumors and aneurysm, is often associated with severe and life-threatening bleeding or organ failure with [1–3]. DIC is characterized by the systemic activation or consumption of components in the coagulation pathways, such as increased fibrin generation or thrombocytopenia, resulting in multiple-organ failure or bleeding tendency [4, 5].

The diagnostic criteria for DIC have been established by the Japanese Ministry of Health and Welfare (JMHW) [6], the International Society of Thrombosis Haemostasis (ISTH) [4], the Japanese Association for Acute Medicine (JAAM) [7] and the Japanese Society of Thrombosis and Hemostasis (JSTH) [8]. These four sets of diagnostic criteria are all based on scoring systems using similar laboratory tests; however, the cutoff values for DIC scores and laboratory test results differ. The JSTH diagnostic criteria [8, 9] classified the diseases underlying DIC into infectious, hematopoietic disorder and basic types.

The guidelines for the diagnosis and treatment of DIC have been published by the British Committee for Standards in Haematology (BCSH) [10], the JSTH [11], the Italian Society for Thrombosis and Haemostasis (SISET) [12] and the ISTH [13]. Most guidelines show that infectious-type DIC is frequently associated with organ failure, whereas hypofibrinogenemia or bleeding tendency is less common. They have recommended the treatment of DIC via treatment of the underlying diseases, along with supportive therapy such as administration of platelet concentrates and fresh frozen plasma [10–13]. The administration of antithrombin (AT) and thrombomodulin alfa (TM-α) are also recommended in the JSTH guidelines [11, 14]. A phase 3 study [15] and several retrospective studies [16–18], including post-marketing surveillance (PMS), have reported the efficacy and safety of TM-α for DIC patients with infectious and hematological diseases. A randomized controlled trial of AT, activated protein C and TM-α for severe sepsis failed to confirm any significant improvement in the outcomes of patients with severe sepsis [19–21].

The present study examined the frequency of hypofibrinogenemia in infectious and hematological diseases with DIC and suspected DIC, and analyzed the characteristics of hypofibrinogenemia in infectious disease patients with DIC.

## Patients and methods

The clinical characteristics and treatment outcomes of patients with DIC and suspected DIC that was treated with TM-α between May 2008 and April 2010 were retrospectively analyzed in a subgroup analysis of PMS data. The original PMS study was an open-label, multicenter, non-interventional, prospective, observational cohort study of patients with DIC who received TM-α [16]. The PMS for TM-α was conducted in accordance with the Japanese Society on Thrombosis and Hemostasis Post-Marketing Surveillance Committee for Recomodulin Injection and the guidelines for Good Postmarketing Study Practice, as required by the Japanese Ministry of Health, Labour, and Welfare (JMHLW). We used existing data without personally identifiable information throughout our study. The original PMS study was therefore exempted from local institutional review and formal approval, as well as the requirement for informed consent. In the PMS for TM-α, all patients were diagnosed with DIC and suspected DIC by attending physicians based on JMHLW or JAAM diagnostic criteria.

The PMS data included 3204 patients with DIC and suspected DIC (patients with infectious diseases, *n* = 2083; patients with hematological diseases, *n* = 1121). The underlying diseases of the patients with infectious diseases were as follows; sepsis (*n* = 837), pneumonia (*n* = 359), peritonitis (*n* = 260), urinary tract infection (*n* = 163), hepatobiliary pancreatic system infection (*n* = 117), skin and muscle infections (*n* = 55), gastrointestinal infections (*n* = 53), meningitis (*n* = 37), viral infection (*n* = 25), wound infection (*n* = 24), pleural infection (*n* = 18), and others (*n* = 135). The underlying diseases of the patients with hematological diseases were as follows; acute myeloid leukemia (*n* = 235), acute promyelocytic leukemia (*n* = 167), non-Hodgkin’s lymphoma (*n* = 156), acute lymphocytic leukemia (*n* = 144), acute myelomonocytic leukemia (*n* = 55), myelodysplastic syndromes (*n* = 51), acute monocytic leukemia (*n* = 47), multiple myeloma (*n* = 35), adult T cell leukemia (*n* = 18), hematopoietic stem cell transplantation (*n* = 17), chronic myelogenous leukemia (n = 17), hemophagocytic syndrome (*n* = 14), acute erythroid leukemia (*n* = 13), febrile neutropenia (*n* = 11), acute megakaryoblastic leukemia (*n* = 7), chronic lymphocytic leukemia (n = 7), aplastic anemia (*n* = 5), Hodgkin’s lymphoma (n = 5), and others (*n* = 117).

The DIC resolution rate, 28-day survival rate, rates of adverse drug reaction and bleeding-related adverse drug reactions were investigated. The degree of coagulopathy was evaluated by calculating DIC scores according to the DIC diagnostic criteria of the JAAM [7] for infectious diseases and those of the JMHW [6] and ISTH [4] for hematological diseases. After treatment with TM-α, resolution of DIC was defined as a score ≤ 3 using the diagnostic criteria of the JAAM, ≤2 using the diagnostic criteria of the JMHW for DIC in patients with hematological diseases, ≤5 using the diagnostic criteria of the JMHW for infectious diseases, and ≤ 4 using the diagnostic criteria of the ISTH for both diseases. The PMS for TM-α started before the establishment of the JSTH DIC diagnostic criteria. As a result, there were few records that included “change of platelet count” or a few data for soluble fibrin and thrombin-antithrombin complex (TAT); thus, the JSTH scores could not be calculated. Survival was calculated at 28 days from the beginning of TM-α treatment or at the end of observation.

In patients with infectious diseases, the severity of organ failure was assessed using the sequential organ failure assessment (SOFA) score [22]. The symptoms of organ failure were decided by the attending physician based on clinical signs indicating organ dysfunction due to DIC [6]. Laboratory tests such as the white blood cell (WBC) count and platelet count, and the measurement of hemoglobin, albumin, lactate dehydrogenase (LDH), total bilirubin (T-Bil), creatine and C-reactive protein (CRP) levels, and hemostatic tests such as the prothrombin time (PT)-international ratio (INR), activated partial thromboplastin time (APTT), fibrinogen, fibrinogen, fibrin degradation products (FDP), D-dimer, AT, protein C, TAT and plasmin-α2 plasmin inhibitor complex (PIC) were performed in each participating institute. Patients with fibrinogen < 1.5 g/L were considered to have hypofibrinogenemia. The rates of adverse drug reaction and bleeding-related adverse drug reaction were evaluated from the start of TM-α treatment to day 28 after the end of TM-α treatment. The safety data were coded using preferred terms from the Japanese translation of the Medical Dictionary for Regulatory Activities (version 13.1).

### Statistical analysis

In the descriptive analyses of baseline characteristics, numerical data were expressed as the median (Q1, Q3; interquartile range). The chi-square test and the Wilcoxon signed-rank test were performed as statistical analyses. *P* values of < 0.05 were considered to indicate statistical significance. Multiplicity adjustment was not considered. All analyses were performed using SAS version 9.4 (SAS Institute, Cary, NC) by EPS Corporation (Tokyo, Japan) according to the plan for the statistical analysis.

## Results

Among cases in which the fibrinogen levels were measured, hypofibrinogenemia (fibrinogen < 1.5 g/L) was observed in 10.3% (215/2083) of the patients with infectious diseases, and 27.8% (312/1121) of the patients with hematological diseases (Table 1). In both diseases, the ratio of males was slightly lower among patients with hypofibrinogenemia than among those without hypofibrinogenemia. Before registration, the administration rate of AT concentrate administration was significantly lower in the infectious disease patients with hypofibrinogenemia than in those without hypofibrinogenemia. The rates of bleeding and organ failure symptoms were both significantly higher in infectious disease patients with hypofibrinogenemia than in those without hypofibrinogenemia. The median SOFA, JMHW, ISTH and JAAM scores were significantly higher in infectious disease patients with hypofibrinogenemia (11, 9, 5 and 6, respectively) than in those without hypofibrinogenemia (10, 6, 4 and 5, respectively; Table 1).

**Table 1:** Demographic characteristics in infectious disease and hematological disease patients with or without hypofibrinogenemia

	Infectious disease					Hematological disease				
Fibrinogen, g/L	≥1.5		< 1.5		*p*-value	≥1.5		< 1.5		p-value
Age, years	1865	71 (60–79)	215	70 (53–79)	0.1094	809	61 (43–70)	311	63 (41–73)	0.0578
Male	1868	1096 (58.7)	215	109 (50.7)	0.0249	809	506 (62.5)	312	165 (52.9)	0.0045
Body weight, kg	1861	50 (40–60)	215	53 (45–62)	0.0005	808.	55 (48.4–64)	312	55 (48.2–63.8)	0.7995
SOFA score	1164	10 (7–13)	118	11 (8–15)	0.0144	–	–	–	–	–
JMHW DIC score	1081	6 (5–7)	104	9 (7–10)	< 0.0001	551	4 (3–5)	226	6 (5–7)	< 0.0001
ISTH DIC score	580	4 (4–5)	54	5 (4–7)	0.0002	209	5 (4–6)	81	6 (5–7)	< 0.0001
JAAM DIC score	1198	5 (4–7)	119	6 (5–8)	0.0005	–	–	–	–	–
Pre-administration	1868		215			809		312		
AT concentrate		364 (19.5)		27 (12.6)	0.0138		62 (7.7)		13 (4.2)	0.0357
Gabexate mesylate		343 (18.4)		42 (19.5)	0.6748		61 (7.5)		29 (9.3)	0.3326
Nafamostat mesylate		175 (9.4)		12 (5.6)	0.0658		29 (3.4)		12 (3.8)	0.8344
Unfractionated heparin		91 (4.9)		6 (2.8)	0.1703		29 (2.4)		7 (2.2)	0.2537
LMWH		34 (1.8)		5 (2.3)	0.6046		65 (8.0)		10 (3.2)	0.0037
Danaparoid sodium		66 (3.5)		8 (3.7)	0.8880		31 (3.8)		20 (6.4)	0.0634
Comorbidities	1868		215			809		312		
Bleeding symptoms		234 (12.5)		54 (25.1)	< 0.0001		225 (27.8)		144 (46.2)	< 0.0001
Organ symptoms		861 (46.1)		116 (54.0)	0.0292		221 (27.3)		71 (22.8)	0.1189

Data are given as n (%) or median (IQR)

DIC, disseminated intravascular coagulation; AT, antithrombin; LMWH, low molecular weight heparin; SOFA, sequential organ failure assessment;

JMHW, Japanese Ministry of Health and Welfare; ISTH, International Society of Thrombosis Haemostasis; JAAM, Japanese Association for Acute Medicine

Regarding the hemostatic examinations, the PT-INR was significantly prolonged among patients with hypofibrinogenemia, and the fibrinogen and AT levels were significantly lower among patients with hypofibrinogenemia than among those without hypofibrinogenemia (**Supplementary Table** **1**). The FDP, D-dimer and TAT levels were significantly higher among patients with hypofibrinogenemia than among those without hypofibrinogenemia. The PIC levels in infectious disease patients with and without hypofibrinogenemia did not differ to a statistically significant extent; however, the PIC levels in hematological disease patients with hypofibrinogenemia were significantly higher than in those without hypofibrinogenemia. The platelet counts in infectious disease patients were significantly lower among patients with hypofibrinogenemia than among those without hypofibrinogenemia (**Supplementary Table** **2**). In both diseases, the albumin and LDH levels were significantly higher and the CRP levels were significantly lower in patients with hypofibrinogenemia than in those without hypofibrinogenemia.

In the treatment of DIC, the TM-α dose and the period of administration in patients with and without hypofibrinogenemia did not differ to a statistically significant extent (**Supplementary Table** **3**). AT concentrate, gabexate mesylate, nafamostat mesylate, unfractionated heparin, low molecular weight heparin, danaparoid sodium, platelet concentrates, fresh frozen plasma and red blood cells were also administered, as shown in **Supplementary Table** **3**. Although the 28-day survival rate of infectious disease patients with hypofibrinogenemia (50.0%) was significantly lower (*p* < 0.0001) than that of infectious disease patients without hypofibrinogenemia (71.6%), the 28-day survival rate of hematological diseases patients with and without hypofibrinogenemia did not differ to a statistically significant extent (Table 2). The resolution rates according to the JMHW, ISTH and JAAM DIC diagnostic criteria were significantly lower (*p* = 0.0058, *p* < 0.0001 and p < 0.0001, respectively) in infectious disease patients with hypofibrinogenemia than in those without hypofibrinogenemia; this difference was not observed in the hematological disease patients with and without hypofibrinogenemia. In both the infectious disease and hematological disease groups, the clinical course of bleeding symptoms did not differ between patients with and without hypofibrinogenemia.

**Table 2:** Outcomes of infectious disease or hematological disease patients in with or without hypofibrinogenemia

	Infectious diseases					Hematological diseases				
Fibrinogen, g/L	≥1.5		< 1.5		p-value	≥1.5		< 1.5		p-value
28-day survival	1846	1321 (71.6)	212	106 (50.0)	< 0.0001	807	587 (72.7)	312	220 (70.5)	0.4566
JMHW DIC resolution	683	427 (62.5)	98	47 (48.0)	0.0058	476	275 (57.8)	231	113 (48.9)	0.0265
ISTH DIC resolution	651	449 (69.0)	119	57 (47.9)	< 0.0001	385	240 (62.3)	202	116 (57.4)	0.2472
JAAM DIC resolution	1266	513 (40.5)	156	30 (19.2)	< 0.000	–	–	–	–	
Clinical course of bleeding symptoms										
Disappeared+Improved	216	99 (45.8)	48	24 (50.0)	0.6007	217	110 (50.7)	142	86 (60.6)	0.0662
Unchanged+Exacerbated	216	117 (54.2)	48	24 (50.0)		217	107 (49.3)	142	56 (39.4)	

Data are given as n (%)

DIC, disseminated intravascular coagulation; JMHW, Japanese Ministry of Health and Welfare; ISTH, International Society of Thrombosis Haemostasis;

JAAM, Japanese Association for Acute Medicine

## Discussion

As hypofibrinogenemia is frequently related to the bleeding tendency in DIC patients with hematological disease and organ failure is often associated with DIC patients with infectious disease, hypofibrinogenemia is considered to be frequently associated with DIC in patients with hematological disease, but rarely with DIC in patients with infectious disease [5, 14]. However, some septic cases have been reported to be associated with severe bleeding [23, 24]. Our analysis of the PMS data revealed that hypofibrinogenemia was present in 10.3% of infectious diseases with DIC and suspected DIC, suggesting that hypofibrinogenemia is sometimes associated with DIC in infectious diseases. As the DIC scores were high, many patients in this study were considered to meet the definition of severe DIC. Thus, the frequency of hypofibrinogenemia in DIC patients with infectious disease may generally be less than 10.3%. Both the frequency of bleeding and organ failure symptoms, and SOFA, JMHW, ISTH and JAAM scores in infectious disease were significantly higher in DIC patients with hypofibrinogenemia than in those without hypofibrinogenemia. This suggests that hypofibrinogenemia in DIC patients with infectious disease can be considered to indicate severe and advanced DIC, in which patients exhibit both bleeding and organ-failure symptoms.

The platelet count in infectious diseases was lower in DIC patients with hypofibrinogenemia than in those without hypofibrinogenemia, suggesting that more severe thrombocytopenia contributes to increased bleeding symptoms or is reflected by multiple thrombi and a subsequent poor prognosis.

The APTT, AT, FDP, D-dimer and TAT levels were significantly greater in patients with hypofibrinogenemia than in those without. In DIC patients with infectious disease, no significant differences in PIC levels were seen between the subgroups with and without hypofibrinogenemia. However, in hematological disease patients with DIC, the PIC levels were significantly higher in patients with hypofibrinogenemia than in those without. These findings indicate that the mechanisms of hypofibrinogenemia in DIC may differ between patients with infectious diseases and hematological diseases, in whom DIC often causes hyperfibrinolysis due to hyper-plasminogen activation by leukemic cells [25, 26]. Conversely, the relatively low levels of PIC in infectious disease did not show hyperfibrinolysis in DIC patients with hypofibrinogenemia, and the increased FDP and D-dimer levels showed the progression of DIC, resulting in high SOFA scores in patients with this type of DIC.

Reduced AT levels may be caused by decreased production due to liver dysfunction, the consumption of AT, leakage into the third space due to increased permeability of vascular endothelial cells [27] or less administration of AT. Although a high DIC score indicates the existence of consumption coagulopathy in DIC patients with severe AT deficiency, decreased albumin and cholinesterase levels and increased T-Bil and creatinine levels suggest that liver dysfunction, plasma leakage, or renal dysfunction might play some role in AT deficiency in patients with DIC. In comparison, the mechanisms underlying hypofibrinogenemia in patients with infectious diseases and DIC are suggested to involve a progressive hypercoagulable state that disseminates and causes the consumption of coagulation factors, such as fibrinogen.

Before registration, the frequency of AT administration was significantly lower in infectious disease patients with hypofibrinogenemia than in those without, suggesting that single anticoagulation therapy tended to be selected in daily clinical practice. Another possibility is that DIC in patients with infectious diseases may result in hypofibrinogenemia with low AT levels in patients who are not treated with AT. The effect of recombinant thrombomodulin administration in sepsis-induced coagulopathy in the SCARLET study [21] was not statistically significant. However, a post hoc analysis revealed a 5.4% reduction in absolute mortality among patients who fulfilled the entry criterion at baseline [28]. Although no significant differences in the TM-α dose or the period of administration were seen between these two states in both types of DIC, the 28-day survival rate and resolution rate according to JMHW, ISTH or JAAM diagnostic criteria in infectious diseases were significantly lower in DIC patients with hypofibrinogenemia than in DIC patients without hypofibrinogenemia. This was only observed in DIC patients with infectious disease. As the mechanism of hypofibrinogenemia in DIC differs between infectious and hematological diseases, hypofibrinogenemia in DIC is considered to be associated with a high risk of infectious diseases, but not hematological diseases. That is, hypofibrinogenemia in infectious diseases may correlate with the severity of DIC, but may be caused by hyperfibrinolysis in hematological diseases, and thus is not associated with poor outcomes. Conversely, no significant differences in the 28-day survival rate or the resolution rate (according to the JMHW, ISTH or JAAM diagnostic criteria for DIC) were evident in hematological disease patients with DIC. DIC patients with increased fibrinogen levels were previously reported to show poor outcomes; however, that previous study included many patients with hematological diseases [29]. High fibrinogen levels might be associated with a hypofibrinolytic state and elevated levels of plasminogen activator inhibitor 1 [30], which is a biomarker for a poor outcome or organ failure due to infection. Production of fibrinogen is upregulated by the liver in infectious diseases, resulting in the observation of increased fibrinogen levels in many DIC patients with infectious diseases [3]. However, hypofibrinogenemia is sometimes caused by consumption of fibrinogen under conditions of hypercoagulability, suppressed production by liver injury, or hyperfibrinolysis [3]. After improvement of these conditions, the plasma fibrinogen level will increase and show various levels depending on the clinical course of infectious disease or liver function. Hypofibrinogen levels may thus strongly reflect the clinical course of the underlying disease, liver function, fibrinolysis, or timing of evaluations [5]. PMS data for TM-α did not show details of bleeding or organ failure symptoms related to the mortality in infectious disease patients with hypofibrinogenemia. Further studies to examine the relationships between severe bleeding or organ failure with hypofibrinogenemia and poor outcome in infectious disease patients with DIC are needed.

In conclusion, DIC in infectious diseases is sometimes associated with hypofibrinogenemia, which reflects the increased consumption of fibrinogen due to accelerated coagulation. Hypofibrinogenemia in DIC patients with infectious diseases is therefore associated with poor outcomes, and the monitoring of fibrinogen levels appears important in the management of DIC in infectious diseases.

## Supplementary Information

**Additional file 1.**

## Data Availability

The data supporting the findings of this study are available from Asahi Kasei Pharma Corporation; however, restrictions apply to the availability of these data, which were used under license for the current study, and so are not publicly available.

## Abbreviations

DIC: Disseminated intravascular coagulation
TM-α: Thrombomodulin alfa
JMHW: Japanese Ministry of Health and Welfare
ISTH: International Society of Thrombosis Haemostasis
JAAM: Japanese Association for Acute Medicine
JSTH: Japanese Society of Thrombosis and Hemostasis
BCSH: British Committee for Standards in Haematology
SISET: Italian Society for Thrombosis and Haemostasis
AT: Antithrombin
PMS: Post-marketing surveillance
JMHLW: Japanese Ministry of Health, Labour, and Welfare
TAT: Thrombin-antithrombin complex
SOFA: Sequential organ failure assessment
WBC: White blood cell
LDH: Lactate dehydrogenase
T-Bil: Total bilirubin
CRP: C-reactive protein
PT-INR: Prothrombin time international normalized ratio
APTT: Activated partial thromboplastin time
FDP: Fibrin and fibrinogen degradation products
PIC: Plasmin-α2 plasmin inhibitor complex
