# Supplementary material for: Hypofibrinogenemia is associated with a high degree of risk in infectious diseases: a post-hoc analysis of post-marketing surveillance of patients with disseminated intravascular coagulation treated with thrombomodulin alfa
Source: Thromb J. 2021 Feb 25;19:12. doi: 10.1186/s12959-021-00264-z (PMC7908729; doi:10.1186/s12959-021-00264-z)
Supplement: Supplementary file 1 — Additional file 1. [file 12959_2021_264_MOESM1_ESM.docx]

**Supplementary Table 1.** Hemostatic characteristics in infectious disease and hematological disease patients with or without hypofibrinogenemia.

|  | Infectious diseases | | | |  |  | Hematological diseases | | | |  |  |
| --- | --- | --- | --- | --- | --- | --- | --- | --- | --- | --- | --- | --- |
| Fibrinogen, g/L | ≥1.5  (n=1868) | | <1.5  (n=215) | | p-value |  | ≥1.5  (n=809) | | <1.5  (n=312) | | p-value |  |
| PT-INR  n | 1.36 | (1.20–1.61)  1813 | 1.84 | (1.43–2.49)  202 | <0.0001 |  | 1.27 | (1.12–1.46)  787 | 1.36 | (1.21–1.62)  300 | <0.0001 |  |
| APTT, s  n | 45.7 | (36.9–60.8)  1714 | 59.3 | (44.1–78.8)  189 | <0.0001 |  | 35.8 | (30.3–43.8)  777 | 32.9 | (28.1–41.15)  300 | 0.0005 |  |
| Fibrinogen, mg/dL  n | 387 | (275–518)  1868 | 114 | (90–132)  215 | <0.0001 |  | 331 | (227–457)  809 | 101 | (71–124)  312 | <0.0001 |  |
| FDP, μg/mL  n | 24.5 | (12.8–47.8)  1540 | 35.4 | (16–110.4)  169 | <0.0001 |  | 31.1 | (16.2–70)  747 | 58.5 | (28.3–115.7)  289 | <0.0001 |  |
| D-dimer, μg/mL  n | 11.86 | (4.62–25.1)  1441 | 16.2 | (6.6–40.2)  171 | 0.0012 |  | 15.2 | (5.85–33.1)  608 | 22.6 | (7.7–47.9)  231 | 0.0017 |  |
| AT, %  n | 56.0 | (44.0–70.9)  1492 | 45.0 | (27.5–63.1)  172 | <0.0001 |  | 82.0 | (67.0–97.0)  583 | 89.0 | (70.0–109.0)  218 | 0.0007 |  |
| Protein C, %  n | 37.8 | (28.0–49.0)  314 | 28.0 | (18.0–52.4)  37 | 0.1162 |  | 61.5 | (43.0–88.0)  166 | 60.4 | (41.0–88.5)  48 | 0.9062 |  |
| TAT, ng/mL  n | 12.8 | (6.6–23.1)  503 | 17.1 | (7.2–42.7)  58 | 0.0256 |  | 15.1 | (8.4–26.7)  244 | 37.9 | (20.4–61.4)  88 | <0.0001 |  |
| PIC, μg/mL  n | 1.6 | (0.8–2.8)  383 | 2.6 | (0.9–4.0)  45 | 0.2596 |  | 3.5 | (1.8–7.6)  228 | 10.9 | (4.9–14.5)  90 | <0.0001 |  |

Data are given as n (%) or median (IQR).

DIC, disseminated intravascular coagulation; PT-INR, prothrombin time-international normalized ratio; APTT, activated partial thromboplastin time;

FDP, fibrin and fibrinogen degradation products; AT, antithrombin; TAT, thrombin-antithrombin complex; PIC, plasmin-plasmin inhibitor complex.

**Supplementary Table 2.** Laboratory characteristics in infectious disease or hematological disease patients with or without hypofibrinogenemia.

|  | Infectious diseases | | | |  |  | Hematological diseases | | | |  |  |
| --- | --- | --- | --- | --- | --- | --- | --- | --- | --- | --- | --- | --- |
| Fibrinogen, g/L | ≥1.5  (n=1868) | | <1.5  (n=215) | | p-value |  | ≥1.5  (n=809) | | <1.5  (n=312) | | p-value |  |
| WBC count, ×10^4^/μL  n | 1.09 | (0.6–1.68)  1860 | 0.99 | (0.51–1.53)  213 | 0.0894 |  | 0.39 | (0.08–1.59)  806 | 0.64 | (0.2–2.85)  309 | <0.0001 |  |
| Platelet count, ×10^4^/μL  n | 5.9 | (3.4–8.9)  1863 | 5.0 | (3.3–7.4)  215 | 0.0023 |  | 2.9 | (1.5–5.5)  808 | 3.1 | (1.7–5.8)  310 | 0.3048 |  |
| Hemoglobin, g/dL  n | 10.1 | (8.8–11.5)  1831 | 9.75 | (8.3–11.2)  210 | 0.0058 |  | 8.3 | (7.2–9.9)  801 | 8.6 | (7.1–10.4)  308 | 0.1677 |  |
| Albumin, g/dL  n | 2.4 | (2.0–2.8)  1639 | 2.6 | (2.1–2.9)  191 | 0.0009 |  | 3.0 | (2.5–3.5)  640 | 3.4 | (2.8–4.0)  252 | <0.0001 |  |
| LDH, IU/L  n | 325 | (228–496)  1718 | 421 | (267–1138)  200 | <0.0001 |  | 458 | (250–1064)  779 | 612 | (346–1295)  297 | <0.0001 |  |
| ChE, IU/L  n | 113 | (69–158)  859 | 98 | (50–168)  99 | 0.3527 |  | 169 | (106–242)  298 | 211 | (128–288)  122 | 0.2689 |  |
| Total bilirubin, mg/dL  n | 1.15 | (0.7–2.27)  1805 | 1.8 | (0.7–2.27)  206 | <0.0001 |  | 0.8 | (0.5–1.6)  755 | 0.8 | (0.5–1.3)  289 | 0.1251 |  |
| Creatine, mg/dL  n | 1.36 | (0.8–2.4)  1850 | 1.31 | (0.82–2.26)  211 | 0.7032 |  | 0.85 | (0.62–1.21)  794 | 0.77 | (0.56–1.02)  304 | 0.0002 |  |
| CRP, mg/dL  n | 17.1 | (10.6–24.1)  1805 | 4.66 | (1.75–9.06)  200 | <0.0001 |  | 7.80 | (2.66–16.7)  731 | 1.50 | (0.31–3.96)  282 | <0.0001 |  |

Data are given as n (%) or median (IQR).

DIC, disseminated intravascular coagulation; WBC, white blood cell; LDH, lactate dehydrogenase; ChE, cholinesterase; CRP, C-reactive protein.

**Supplementary Table 3.** DIC therapy in infectious disease or hematological disease patients with or without hypofibrinogenemia.

|  | Infectious diseases | | | |  |  | Hematological diseases | | | |  |  |
| --- | --- | --- | --- | --- | --- | --- | --- | --- | --- | --- | --- | --- |
| Fibrinogen, g/L | ≥1.5  (n=1868) | | <1.5  (n=215) | | p-value |  | ≥1.5  (n=809) | | <1.5  (n=312) | | p-value |  |
| TM-α administration |  |  |  |  |  |  |  |  |  |  |  |  |
| Dose, U/day  n | 372 | (183–380)  1861 | 361 | (139–380)  215 | 0.3769 |  | 380 | (347–380)  808 | 380 | (360–381)  312 | 0.2437 |  |
| Period, days  n | 6 | (4–6)  1868 | 6 | (4–7)  215 | 0.7167 |  | 6 | (5–8)  809 | 6 | (5–7.5)  312 | 0.5312 |  |
| Concomitant |  |  |  |  |  |  |  |  |  |  |  |  |
| AT concentrate | 941 | (50.4) | 119 | (55.3) | 0.1671 |  | 172 | (21.3) | 49 | (15.7) | 0.0361 |  |
| Gabexate mesylate | 599 | (32.1) | 53 | (24.7) | 0.0264 |  | 63 | (7.8) | 36 | (11.5) | 0.0473 |  |
| Nafamostat mesylate | 410 | (21.9) | 40 | (18.6) | 0.2592 |  | 46 | (5.7) | 14 | (4.5) | 0.4241 |  |
| Unfractionated heparin | 311 | (16.6) | 42 | (19.5) | 0.2854 |  | 136 | (16.8) | 42 | (13.5) | 0.1691 |  |
| LMWH | 61 | (3.3) | 2 | (0.9) | 0.0583 |  | 63 | (7.8) | 6 | (1.9) | 0.0003 |  |
| Danaparoid sodium | 128 | (6.9) | 16 | (7.4) | 0.7469 |  | 36 | (4.4) | 15 | (4.8) | 0.7967 |  |
| Platelet concentrate | 408 | (21.8) | 60 | (27.9) | 0.0436 |  | 351 | (43.4) | 152 | (48.7) | 0.1078 |  |
| Fresh frozen plasma | 368 | (19.7) | 89 | (41.4) | <0.0001 |  | 110 | (13.4) | 142 | (45.5) | <0.0001 |  |
| Red blood cells | 412 | (22.1) | 70 | (32.6) | 0.0005 |  | 295 | (36.5) | 118 | (37.8) | 0.6732 |  |

Data are given as n (%) or median (IQR).

DIC, disseminated intravascular coagulation; TM-α, thrombomodulin alfa; AT, antithrombin; LMWH, low molecular weight heparin.
